# Predicting neurological outcome in adult patients with cardiac arrest: systematic review and meta-analysis of prediction model performance

**DOI:** 10.1186/s13054-022-04263-y

**Published:** 2022-12-11

**Authors:** Simon A. Amacher, René Blatter, Matthias Briel, Christian Appenzeller-Herzog, Chantal Bohren, Christoph Becker, Katharina Beck, Sebastian Gross, Kai Tisljar, Raoul Sutter, Stephan Marsch, Sabina Hunziker

**Affiliations:** 1 Intensive Care, University Hospital Basel, Basel, Switzerland; 2 Medical Communication and Psychosomatic Medicine, University Hospital Basel, Klingelbergstrasse 23, 4031 Basel, Switzerland; 3 Meta-Research Centre, Department of Clinical Research, University of Basel and University Hospital Basel, Basel, Switzerland; 4 Department of Health Research Methodology, Evidence, and Impact, McMaster University, Hamilton, Canada; 5 Medical Faculty, University of Basel, Basel, Switzerland; 6 University Medical Library, University of Basel, Basel, Switzerland; 7 Department of Emergency Medicine, University Hospital Basel, Basel, Switzerland

**Keywords:** Cardiac arrest, CAHP, OHCA, GO-FAR, Neurological outcome, Prognostication, Prediction model

## Abstract

This work aims to assess the performance of two post-arrest (out-of-hospital cardiac arrest, OHCA, and cardiac arrest hospital prognosis, CAHP) and one pre-arrest (good outcome following attempted resuscitation, GO-FAR) prediction model for the prognostication of neurological outcome after cardiac arrest in a systematic review and meta-analysis. A systematic search was conducted in Embase, Medline, and Web of Science Core Collection from November 2006 to December 2021, and by forward citation tracking of key score publications. The search identified 1′021 records, of which 25 studies with a total of 124′168 patients were included in the review. A random-effects meta-analysis of C-statistics and overall calibration (total observed vs. expected [O:E] ratio) was conducted. Discriminatory performance was good for the OHCA (summary C-statistic: 0.83 [95% CI 0.81–0.85], 16 cohorts) and CAHP score (summary C-statistic: 0.84 [95% CI 0.82–0.87], 14 cohorts) and acceptable for the GO-FAR score (summary C-statistic: 0.78 [95% CI 0.72–0.84], five cohorts). Overall calibration was good for the OHCA (total O:E ratio: 0.78 [95% CI 0.67–0.92], nine cohorts) and the CAHP score (total O:E ratio: 0.78 [95% CI 0.72–0.84], nine cohorts) with an overestimation of poor outcome. Overall calibration of the GO-FAR score was poor with an underestimation of good outcome (total O:E ratio: 1.62 [95% CI 1.28–2.04], five cohorts). Two post-arrest scores showed good prognostic accuracy for predicting neurological outcome after cardiac arrest and may support early discussions about goals-of-care and therapeutic planning on the intensive care unit. A pre-arrest score showed acceptable prognostic accuracy and may support code status discussions.

**Graphical Abstract:**

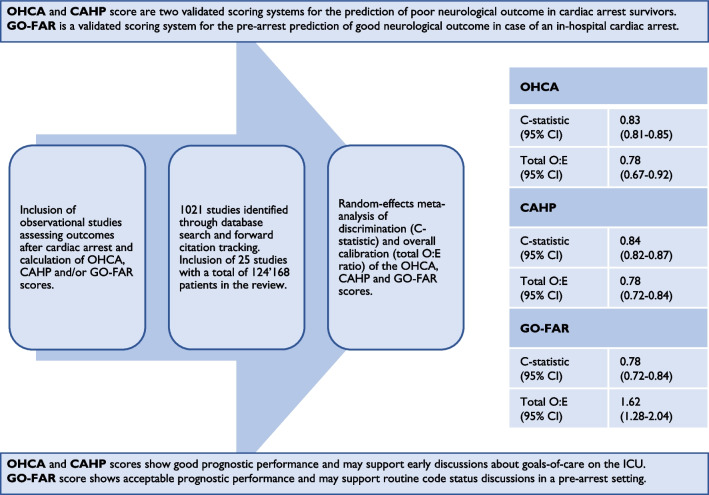

**Supplementary Information:**

The online version contains supplementary material available at 10.1186/s13054-022-04263-y.

## Introduction

Cardiac arrest is a significant cause of premature death worldwide with high mortality and the risk of unfavourable neurological outcome due to hypoxic-ischaemic brain injury [1–5]. Intensive care unit (ICU) physicians frequently encounter severely ill cardiac arrest survivors in a state of persistent reduced consciousness and haemodynamic instability sometimes complicated by sedation and paralysis due to targeted temperature management. These circumstances render prognostication difficult, which could lead to overly pessimistic prognosis and unjustified early withdrawal of life-sustaining therapy (WLST) [6–10]. Thus, current guidelines recommend delaying prognostication to 72 h after return of spontaneous circulation (ROSC) [11]. The uncertainty during the first three days renders early discussions about goals of care and therapeutic planning between physicians and surrogate decision makers (i.e., next-of-kin) difficult.

Post-cardiac-arrest clinical predictive models (CPM) based on patient-specific parameters (e.g., no-flow and low-flow intervals, initial cardiac arrest rhythm, age, arrest setting) could support these early discussions by stratifying patients according to the chance of survival with a good neurological outcome [12]. The Cardiac Arrest Hospital Prognosis (CAHP) score and the Out-of-Hospital Cardiac Arrest (OHCA) score are two well-validated CPM which predict survival to hospital discharge with a good neurological outcome in cardiac arrest survivors as measured by the cerebral performance category scale (CPC) [13–15] (Box 1).

**Box 1:** Description of Included Scores

Score	Outcome predicted	Variable	Score calculation	Probability categories for primary outcome in original publication
OHCA	Poor neurological outcome (CPC 3–5)	Initial rhythm: VF or VT [yes/no]	− 13 if no	2.0: 25% risk
		No-flow interval [min]	+ 6 × ln(no-flow interval)	17.4: 50% risk
		Low-flow interval [min]	+ 9 × ln(low-flow interval)	32.5: 75% risk
		Serum creatinine [µmol/L]	− 1434/(serum creatinine)	
		Arterial lactate [mmol/L]	+ 10 × ln(arterial lactate)	
CAHP	Poor neurological outcome (CPC 3–5)	Age [years]	Points attributed by nomogram for all variables	< 150: low risk
		Arrest Setting [home/public]		150 to 200: medium risk
		Shockable Rhythm [yes/no]		> 200: high risk
		No-flow interval [min]		
		Low-flow interval [min]		
		pH at Admission		
		Dosage of Epinephrine administered [0, 1–2 or ≥ 3 mg]		
GO-FAR	Good neurological outcome (CPC 1)	CPC 1 at admission	− 15	≥ 24: very low
		Major trauma	10	14 to 23: low
		Acute stroke	8	− 5 to 13: average
		Metastatic or hematologic cancer	7	− 15 to − 6: above average
		Septicaemia	7	
		Medical non-cardiac diagnosis	7	
		Hepatic insufficiency	6	
		Admitted from skilled nursing facility	6	
		Hypotension or hypoperfusion	5	
		Renal insufficiency or dialysis	4	
		Respiratory insufficiency	4	
		Pneumonia	1	
		Age 70–74 years	2	
		Age 75–79 years	5	
		Age 80–84 years	6	
		Age ≥ 85 years	11	

*CAHP* Cardiac Arrest Hospital Prognosis; *CPC* Cerebral Performance Category; *GO-FAR* Good Outcome Following Attempted Resuscitation; *OHCA* Out-of-hospital cardiac arrest; *VF* Ventricular fibrillation; *VT* Ventricular tachycardia

Another important application of CPM is to predict outcome after cardiac arrest in a pre-arrest setting, specifically in code status discussions [16, 17]. As in-hospital cardiac arrest (IHCA) is a frequent emergency with potentially devastating outcomes and high health care costs [18, 19], shared decision-making with patients at hospital admission concerning advanced care planning and do-not-attempt-resuscitation orders should be standard of care, especially in elderly polymorbid patients [20–25]. Knowledge about the expected chances of survival with good neurofunctional recovery following a cardiac arrest can guide patients and physicians in this difficult decision-making process. The Good Outcome Following Attempted Resuscitation (GO-FAR) score is a pre-arrest CPM, which has shown potential as a tool to assess futility regarding cardiopulmonary resuscitation (CPR) and might thus be valuable to support code status discussions [16, 17]. It predicts the chance of survival to hospital discharge with a favourable neurological outcome as measured by CPC in case of an in-hospital cardiac arrest [16]. The score is based on a variety of pre-arrest predictors for unfavourable outcome, such as age, comorbidities, organ insufficiency, or pre-admission functional status [16] (Box 1).

A systematic review of available CPM identified the OHCA, CAHP, and GO-FAR scores as the three most thoroughly validated scoring systems for the prediction of neurological outcome after cardiac arrest [12]. A meta-analysis of the predictive performance was not conducted. However, the numerous external validation studies provide a complex overall picture that might be challenging to oversee for the individual bedside physician. Hence, evidence synthesis in the form of a systematic review and meta-analysis is of utmost importance [26]. The present work aims to assess the predictive performance of the three most rigorously validated CPM within the framework of a state-of-the-art systematic review and meta-analysis.

## Methods

Data collection and reporting for this systematic review and meta-analysis followed the Checklist for Critical Appraisal and Data Extraction for Systematic Reviews of Prediction Modelling Studies (CHARMS), the Preferred Reporting Items for Systematic Reviews and Meta-analyses (PRISMA), and the Meta-analysis of Observational Studies in Epidemiology (MOOSE) checklist [27–29]. To avoid duplication, data-driven research, and reporting bias the study protocol was preregistered in the register for systematic reviews PROSPERO (registration no. CRD42022287816).

### Search strategy

The search strings were developed by an information specialist (CAH). The bibliographic databases Embase (Elsevier), Medline (Ovid), and Web of Science Core Collection (Clarivate) were searched using the three score names and their acronyms for studies on adult patients (last search conducted on December 7, 2021). The publication date was restricted to after November 1^st^, 2006, when the original OHCA score study was published [13]. The complete search strategies can be found in the online-only Additional file 1. Furthermore, following an evidence-based methodology [30], the citing references of the three original score publications [13, 14, 16] and their validation studies [31–42], as compiled in a recent survey of current science [12], were downloaded from Scopus and Web of Science. All references were exported to EndNote 20 (Clarivate Analytics, London, United Kingdom) and de-duplicated using the Bramer method [43].

### Study selection

Eligible for this systematic review and meta-analysis were all studies meeting the following inclusion criteria: Observational study design; inclusion of patients admitted to the ICU after in- or out-of-hospital cardiac arrest; assessment of mortality and/or neurological outcome; calculation of the OHCA, CAHP and/or GO-FAR score. Studies were excluded based on publication type (reviews, congress abstracts, comments, case reports, case series, randomised controlled trials, animal studies), language (any language other than English or German), publication date before November 1st, 2006 (publication date of the original OHCA score development study), and if more than 20% of paediatric patients (< 18 years of age) were included.

Two study team members (SAA and RB) conducted the title and abstract screening and the following full-text screening independently following a standardised form with inclusion and exclusion criteria. Disagreements were discussed until consensus was reached. If no consensus could be reached, a final decision was made by the research team leader (SH).

### Outcomes

The primary outcome was neurological outcome assessed by the CPC, including death in accordance with the original publications for each score [13, 14, 16]. The CPC is a well-validated outcome score commonly used to assess neurological function in post-cardiac-arrest patients. It differentiates five levels of neurological functioning: A CPC score of 1 corresponds to survival with no or minor neurological deficits and no impairment of everyday functioning. A CPC score of 2 indicates moderate cerebral disability with impairment of working life, but patients are still able to conduct activities of daily living independently. A CPC score of 3 indicates severe cerebral disability with dependence on support from others for everyday living. A CPC score of 4 stands for patients in a coma or vegetative state, and a CPC score of 5 equals brain death or death [15].

For the OHCA and CAHP score, neurological outcome was defined as good (CPC score 1 to 2) or poor (CPC score 3 to 5) [13, 14]. In accordance with the original publication [16], good neurological outcome for the GO-FAR score was defined as CPC score 1, poor neurological outcome as CPC score 2 to 5. Secondary outcomes were mortality at hospital discharge and at one, three, six, and twelve months.

### Data extraction and handling of missing data

Data extraction was performed in accordance with the CHARMS[28] checklist independently and in duplicate by two members of the study team (SAA and RB). The extraction of prognostic accuracy measures and the handling of missing data followed the methodology recommended by the Prognosis Methods Group of the Cochrane Collaboration [44].

The following information was extracted to assess study characteristics: Study design (i.e., development or validation; prospective or retrospective study design), country, study period, number of participants, inclusion criteria, primary and key secondary outcomes. To assess heterogeneity between studies, data concerning differences in baseline characteristics of the populations (i.e., age, sex, cardiac arrest aetiology) and the observed percentage of good outcome was extracted as suggested by Debray et al.[45]

To assess the performance of the prognostic scores, measures of discrimination, calibration, and classification were extracted [28]. To assess discrimination, the area under the receiver operating characteristic curve (AUROC, C-statistic) with corresponding uncertainty measures were extracted and missing uncertainty measures estimated using the approach developed by Debray et al.[45]

To assess calibration, the total observed vs. expected (O:E) ratio was analysed [45]. Observed and expected rates of poor neurological outcome were extracted for the total cohort and if possible for each risk stratum. In case of missing expected outcomes, missing values were approximated using two evidence-based approaches: To calculate the O:E ratio for the GO-FAR score, the approach proposed and validated by Dimitrov et al.[46] was applied. Briefly, the outcome frequencies in the original development cohort for each risk stratum were extracted and applied to the validation cohort to calculate expected outcome numbers per risk stratum. To calculate the missing expected outcome rates for the OHCA and CAHP scores, the mean or median values of the patient characteristics were incorporated into the prediction model to calculate an overall mean score value for the population in the validation study. The score value was then transformed into a probability of poor outcome. For the OHCA score, this could be achieved using a formula published in the original publication [13], for the CAHP score, the original nomogram[14] was used. If reported, the mean or median score values were extracted directly. The probability derived from the score value was then applied to the validation cohort to obtain the expected outcome number. The variance of the total O:E ratios was calculated on the log scale using the equations provided by Debray et al. [45]

Classification measures (i.e., sensitivity, specificity, positive predictive value, and negative predictive value) at specific score cut-offs were extracted as reported.

### Risk of bias assessment

The risk of bias (ROB) was assessed using the Prediction Model Risk of Bias Assessment Tool (PROBAST) [47], which evaluates ROB in the following four domains: Participants, predictors, outcome, and analysis. Additionally, it assesses the applicability of each study to the review question in three domains: Participants, predictors and outcome. Specific signalling questions help to identify potential sources of ROB or non-applicability. ROB and applicability rating were conducted independently and in duplicate by two study team members (SAA and RB). Cases of disagreement were discussed until consensus was reached.

### Statistical analysis

Only external validation cohorts were included in the meta-analysis of the prognostic performance measures [45]. Meta-analysis of C-statistics and total O:E ratios was following a validated approach recommended by the Prognosis Methods Group of the Cochrane Collaboration [44, 45]. Meta-analysis of C-statistics was conducted using a random-effects model with restricted maximum likelihood (REML) estimation using the metaan procedure in STATA 15 (StataCorp, College Station, Texas, United States). A C-statistic of 0.7–0.8 was defined as acceptable, 0.8–0.9 as good and > 0.9 as excellent. Between-study normality of C-statistics was assessed visually prior to the analysis. A second meta-analysis on the logit scale was conducted to check the validity of the results. This approach ensures normal distribution of C-statistics between studies [45, 48]. Heterogeneity was estimated using the I^2^ statistic. Meta-analysis of total O:E ratios was conducted on the log scale using the random-effects model with REML estimation as described above [45, 48].

To assess the prognostic performance of the scores using the risk strata with the cut-offs defined in the original publications [13, 14, 16], the approach suggested by Ebell et al.[49] was applied to obtain stratum-specific likelihood ratios.

A pre-specified subgroup analysis was conducted comparing studies assessing outcomes after OHCA only versus studies evaluating outcomes after IHCA or in samples with both IHCA and OHCA patients. Additionally, subgroup analyses not pre-specified in the protocol were performed to address heterogeneity in the outcome assessment across the validation studies. Subgroup analyses were conducted comparing studies assessing neurological outcome versus those assessing mortality only and studies assessing outcome at hospital discharge to 30 days versus those assessing outcome more than 1 month after the cardiac arrest.

Two separate meta-regression analyses were conducted to assess if heterogeneity in C-statistics between validation studies can partly be explained by heterogeneity in specific characteristics of the respective cohorts. The C-statistic of the validation studies was included as the dependent variable. As the independent variable, the observed percentage of good outcome in the validation study and mean/median patient age were included.

## Results

### Study selection process

**Fig. 1:**
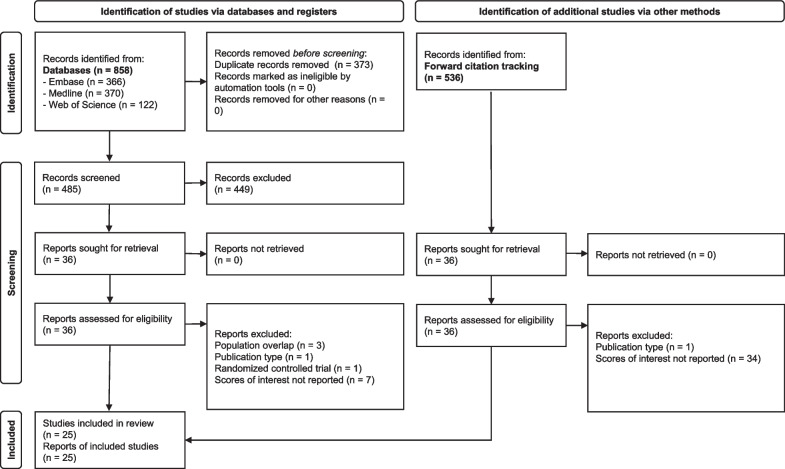
Flowchart of the search and screening process

A total of 1′021 unique records were identified through database searches (*n* = 485) and forward citation tracking (*n* = 536) and screened on titles and abstracts. Figure 1 outlines the study selection process [27]. A total of 72 selected records were screened in full text, of which 25 records with 124′168 patients were included in the final review and summarised in Table 1.

**Table 1:** Characteristics of included studies

Author	Year	Study design	Sample size	Inclusion criteria	Exclusion criteria	Time of outcome assessment	Primary outcome	Key secondary outcomes	Comments
OHCA score									
Adrie et al. (development cohort)	2006	PCS single-centre	130	OHCA	Age < 18 years	Hospital discharge	Poor neurological outcome (CPC > 2)	None	
Adrie et al. (validation cohort)	2006	PCS multicentre	210	OHCA	Age < 18 years	Hospital discharge	Poor neurological outcome (CPC > 2)	None	
Hunziker et al	2011	RCS bi-centre	128	OHCA	Age < 18 years; traumatic aetiology	Hospital discharge	Poor neurological outcome (CPC > 2)	None	
Skrifvars et al	2012	PCS single-centre	122	OHCA and IHCA	ROSC without chest compressions	Hospital discharge	Mortality	ICU mortality, mortality at 1 month	
Bisbal et al	2014	RCS single-centre	124	OHCA and IHCA	Age < 18 years; death within 1 h after ROSC	Hospital discharge	Mortality	Poor neurological outcome (CPC > 2) at ICU discharge, 3, 6 and 12 months	Primary aim: Validation of SAPS III score
Sauneuf et al	2016	PCS single-centre	204	OHCA	Age < 18 years; pregnancy; immunosuppression	Hospital discharge	Poor neurological outcome (CPC > 2)	None	Primary aim: Development of MyeloScore
Choi et al	2018	RCS single-centre	173	OHCA treated with TTM	Traumatic aetiology	1 month	Poor neurological outcome (CPC > 2)	None	
Isenschmid et al	2019	PCS single-centre	349	OHCA and IHCA	Age < 16 years; monitored IHCA	Hospital discharge	Mortality	Poor neurological outcome (CPC > 2) at hospital discharge, mortality at 1 month	
Chelly et al	2020	RCS multicentre	381	IHCA	Age < 18 years; GCS > 8; monitored IHCA	Hospital discharge	Poor neurological outcome (CPC > 2)	None	
Kim et al	2020	RCS single-centre	311	OHCA treated with TTM	None	Hospital discharge	Poor neurological outcome (CPC > 2)	None	
Pareek et al. (KOCAR cohort)	2020	PCS multicentre	373	OHCA	Age < 18 years; non-cardiac aetiology; intracranial bleeding; CPC > 2 prior to admission; life-expectancy < 6 months; GCS 15 at admission	6 months	Poor neurological outcome (CPC > 2)	None	Primary aim: Development of MIRACLE2 score
Pareek et al. (Ljubljana cohort)	2020	PCS single-centre	325	OHCA	Age < 18 years; non-cardiac aetiology; intracranial bleeding; CPC > 2 prior to admission; life-expectancy < 6 months; GCS 15 at admission	Hospital discharge	Poor neurological outcome (CPC > 2)	None	Primary aim: Validation of MIRACLE2 score
Pareek et al. (RFH cohort)	2020	PCS single-centre	148	OHCA	Age < 18 years; non-cardiac aetiology; intracranial bleeding; CPC > 2 prior to admission; life-expectancy < 6 months; GCS 15 at admission	6 months	Poor neurological outcome (CPC > 2)	None	Primary aim: Validation of MIRACLE2 score
Bae et al	2021	RCS single-centre	297	OHCA	Age < 18 years; traumatic aetiology; stroke; unwitnessed OHCA; CPC 2 prior to admission	Hospital discharge	Poor neurological outcome (CPC > 2)	None	Primary aim: Development and validation of PROLOGUE score; no WLST allowed unless patients were pronounced brain dead
Pham et al	2021	PCS single-centre	386	OHCA due to ACS treated with PCI	None	Hospital discharge	Mortality	None	
Shibahashi et al	2021	PCS multicentre	2428	OHCA	Age < 18 years; extrinsic aetiology (trauma, burn, hypothermia, drowning, asphyxiation, intoxication)	1 month	Poor neurological outcome (CPC > 2)	None	Primary aim: Validation of sOHCA and sCAHP scores
Song et al	2021	RCS single-centre	106	OHCA treated with TTM	Age < 18 years; traumatic aetiology; ineligibility for TTM; TTM induced > 6 h after ROSC; failure to maintain target temperature	3 months	Poor neurological outcome (CPC > 2)	None	
Tsuchida et al	2021	RCS single-centre	189	OHCA	Age < 18 years; transfers from other hospitals	1 month	Poor neurological outcome (CPC > 2)	None	
CAHP score									
Maupain et al. (development cohort)	2016	PCS multicentre	819	OHCA	Extrinsic aetiology (trauma, hypothermia, drowning, asphyxiation, intoxication)	ICU discharge	Poor neurological outcome (CPC > 2)	None	
Maupain et al. (internal validation cohort)	2016	PCS multicentre	1129	OHCA	Extrinsic aetiology (trauma, hypothermia, drowning, asphyxiation, intoxication)	ICU discharge	Poor neurological outcome (CPC > 2)	None	
Maupain et al. (external validation cohort)	2016	RCS single-centre	367	OHCA	Extrinsic aetiology (trauma, hypothermia, drowning, asphyxiation, intoxication)	ICU discharge	Poor neurological outcome (CPC > 2)	None	
Sauneuf et al	2016	PCS single-centre	204	OHCA	Age < 18 years; pregnancy; immunosuppression	Hospital discharge	Poor neurological outcome (CPC > 2)	None	Primary aim: Development of the MyeloScore
Isenschmid et al	2019	PCS single-centre	349	OHCA and IHCA	Age < 16 years; monitored IHCA	Hospital discharge	Mortality	Poor neurological outcome (CPC > 2) at hospital discharge, mortality at 1 month	
Chelly et al	2020	RCS multicentre	381	IHCA	Age < 18 years; GCS > 8; monitored IHCA	Hospital discharge	Poor neurological outcome (CPC > 2)	None	
Kim et al	2020	RCS single-centre	311	OHCA treated with TTM	None	Hospital discharge	Poor neurological outcome (CPC > 2)	None	
Pareek et al. (KOCAR cohort)	2020	PCS multicentre	373	OHCA	Age < 18 years; non-cardiac aetiology; intracranial bleeding; CPC > 2 prior to admission; life-expectancy < 6 months; GCS 15 at admission	6 months	Poor neurological outcome (CPC > 2)	None	Primary aim: Development of MIRACLE2 score
Pareek et al. (Ljubljana cohort)	2020	PCS single-centre	325	OHCA	Age < 18 years; non-cardiac aetiology; intracranial bleeding; CPC > 2 prior to admission; life-expectancy < 6 months; GCS 15 at admission	Hospital discharge	Poor neurological outcome (CPC > 2)	None	Primary aim: Validation of MIRACLE2 score
Pareek et al. (RFH cohort)	2020	PCS single-centre	148	OHCA	Age < 18 years; non-cardiac aetiology; intracranial bleeding; CPC > 2 prior to admission; life-expectancy < 6 months; GCS 15 at admission	6 months	Poor neurological outcome (CPC > 2)	None	Primary aim: Validation of MIRACLE2 score
Sauneuf et al	2020	RCS multicentre	176	OHCA in patients > 75 years	Non-French speaking patients; traumatic aetiology; conscious patients	Hospital discharge	Poor neurological outcome (CPC > 2)	Poor neurological outcome (CPC > 2) at 6 and 12 months	
Bae et al	2021	RCS single-centre	297	OHCA	Age < 18 years; traumatic aetiology; stroke; unwitnessed OHCA; CPC > 2 prior to admission	Hospital discharge	Poor neurological outcome (CPC > 2)	None	Primary aim: Development and validation of PROLOGUE score; no WLST allowed unless patients were pronounced brain dead
Pham et al	2021	PCS single-centre	386	OHCA due to ACS treated with PCI	None	Hospital discharge	Mortality	None	
Shibahashi et al	2021	PCS multicentre	2428	OHCA	Age < 18 years; extrinsic aetiology (trauma, burn, hypothermia, drowning, asphyxiation, intoxication)	1 month	Poor neurological outcome (CPC > 2)	None	Primary aim: Validation of sOHCA and sCAHP scores
Song et al	2021	RCS single-centre	106	OHCA treated with TTM	Age < 18 years; traumatic aetiology; ineligibility for TTM; TTM induced > 6 h after ROSC; failure to maintain target temperature	3 months	Poor neurological outcome (CPC > 2)	None	
Tsuchida et al	2021	RCS single-centre	189	OHCA	Age < 18 years; transfers from other hospitals	1 month	Poor neurological outcome (CPC > 2)	None	
Vedamurthy et al	2021	RCS single-centre	158	OHCA	Extrinsic aetiology (trauma, asphyxiation, pulmonary embolism, exacerbated chronic lung disease)	Hospital discharge	Mortality	Poor neurological outcome (CPC > 2) at hospital discharge	
GO-FAR score									
Ebell et al. (development and internal validation cohort)	2013	PCS multicentre	51,240	IHCA	None	Hospital discharge	Good neurological outcome (CPC = 1)	None	Cohort randomly divided into development, training and test data sets
Ohlsson et al	2016	RCS single-centre	287	IHCA	Age < 18 years	Hospital discharge	Good neurological outcome (CPC = 1)	Mortality	
Piscator et al	2018	RCS multicentre	717	IHCA	Age < 18 years	Hospital discharge	Good neurological outcome (CPC = 1)	None	
Rubins et al	2019	RCS single-centre	403	IHCA	Age < 18 years	Hospital discharge	Good neurological outcome (CPC = 1)	Mortality	
Thai et al	2019	PCS multicentre	62,131	IHCA	Age < 18 years	Hospital discharge	Good neurological outcome (CPC = 1)	None	
Cho et al	2020	RCS single-centre	1011	IHCA	Age < 18 years; major trauma	Hospital discharge	Good neurological outcome (CPC < 3)	Good neurological outcome (CPC = 1)	
Aldabagh et al	2021	RCS single-centre	884	IHCA	Age < 18 years; transfers from other hospitals; patients who changed their status to do-not-resuscitate after survived IHCA	Hospital discharge	Mortality	None	Included 60.2% COVID-19 patients

*ACS* Acute coronary syndrome; *CAHP* Cardiac Arrest Hospital Prognosis; *COVID-19* Coronavirus disease 2019; *CPC* Cerebral Performance Category; *GCS* Glasgow Coma Scale; *GO-FAR* Good Outcome Following Attempted Resuscitation; *ICU* Intensive care unit; *IHCA* In-hospital cardiac arrest; *KOCAR* King’s Out-of-Hospital Cardiac Arrest Registry; *OHCA* Out-of-hospital cardiac arrest; *PCI* Percutaneous coronary intervention; *PCS* Prospective cohort study; *RCS* Retrospective cohort study; *RFH* Royal Free Hospital London; *ROSC* Return of spontaneous circulation; *TTM* Targeted temperature management; *WLST* Withdrawal of life-sustaining therapy

### Characteristics of included studies: OHCA score

Fifteen studies [13, 31, 32, 34, 35, 50–59] reporting outcomes from 18 cohorts (ten prospective and eight retrospective cohorts) with a total of 4′747 patients were included in the review, of which 16 external validation cohorts [31, 32, 34, 35, 50–59] were available for the evaluation of the OHCA score’s prognostic performance. The studies were mainly performed in Europe (*n* = 7) [13, 35, 50, 52, 54, 56, 60] and in Asia (*n* = 6) [34, 53, 55, 57–59] with one study each performed in the USA[31] and Australia [32], respectively. The majority of studies (*n* = 11) [13, 31, 34, 51, 53–59] reported outcomes after OHCA, whereas three studies [32, 35, 50] reported outcomes of both OHCA and IHCA patients, and one study [52] reported outcomes after IHCA only. In accordance with the original publication of the OHCA score [13], the majority of studies assessed the prognostic performance of the OHCA score for the prediction of neurological outcome (*n* = 12) [13, 31, 34, 35, 51–55, 57–59]. In contrast, three studies[32, 50, 56] assessed the score’s performance in predicting mortality only. The outcome assessment was performed at hospital discharge or 30 days in 15 cohorts[13, 31, 32, 34, 35, 50–57, 59] and at > 1 month in three cohorts [54, 58] (Box 1).

### Characteristics of included studies: CAHP score

Thirteen studies [14, 35, 38, 51–59, 61] reporting outcomes from 17 cohorts (nine prospective and eight retrospective cohorts) with a total of 6′769 patients were included in the review, of which 14 external validation cohorts [14, 35, 38, 51–59, 61] were available to evaluate the CAHP score’s prognostic performance. The studies were mainly performed in Europe (*n* = 7) [14, 35, 38, 51, 52, 54, 56] and in Asia (*n* = 5) [53, 55, 57–59], with only one study [61] performed in the USA. The majority of studies (*n* = 11) [14, 38, 51, 53–59, 61] reported outcomes after OHCA, whereas one study [35] reported outcomes of mixed OHCA and IHCA patients and one study [52] after IHCA only. In accordance with the original publication of the CAHP score [14], the majority of studies assessed the prognostic performance of the CAHP score for the prediction of neurological outcome (*n* = 12), while only one study [57] assessed the score’s performance in predicting mortality only. The outcome assessment was performed at hospital discharge or 30 days in 13 cohorts [14, 35, 38, 51–57, 59, 61] and at > 1 month in three cohorts [54, 58].

### Characteristics of included studies: GO-FAR score

Seven studies [16, 40–42, 62–64] reporting outcomes from seven cohorts (two prospective and five retrospective cohorts) with a total of 116′673 patients were included in the review, of which five external validation cohorts [40–42, 62, 63] were available for the evaluation of the GO-FAR score’s prognostic performance. The studies were performed in the USA (*n* = 4) [16, 41, 42, 64], in Europe (*n* = 2) [40, 62] and Asia (*n* = 1) [63]. In accordance with the original publication of the GO-FAR score, the vast majority of studies assessed the prognostic performance of the GO-FAR score for the prediction of neurological outcome (*n* = 6) [16, 40–42, 62, 63], while one study [64] assessed the score’s performance in predicting mortality only. The outcome was assessed at hospital discharge in all cohorts.

### Risk of bias

Twenty-three out of 25 studies [13, 14, 16, 31, 32, 34, 35, 38, 40–42, 50–56, 58, 59, 61, 63, 64] were found to be at high risk of bias (Additional file 1: Table S1). The high risk of bias ratings were due to issues in the “analysis” domain. The following three main issues were identified: First, the failure to include an appropriate number of patients (defined as at least 100 participants with the less frequent outcome event) [47, 65, 66]. Second, the inappropriate handling of missing data, if studies either excluded a substantial percentage of patients with missing outcome data from the analysis or if missing predictor data was not handled using multiple imputation. Third, most of the studies omitted to report calibration measures appropriately (Additional file 1: Table S2).

### Prognostic performance of the OHCA score

**Fig. 2:**
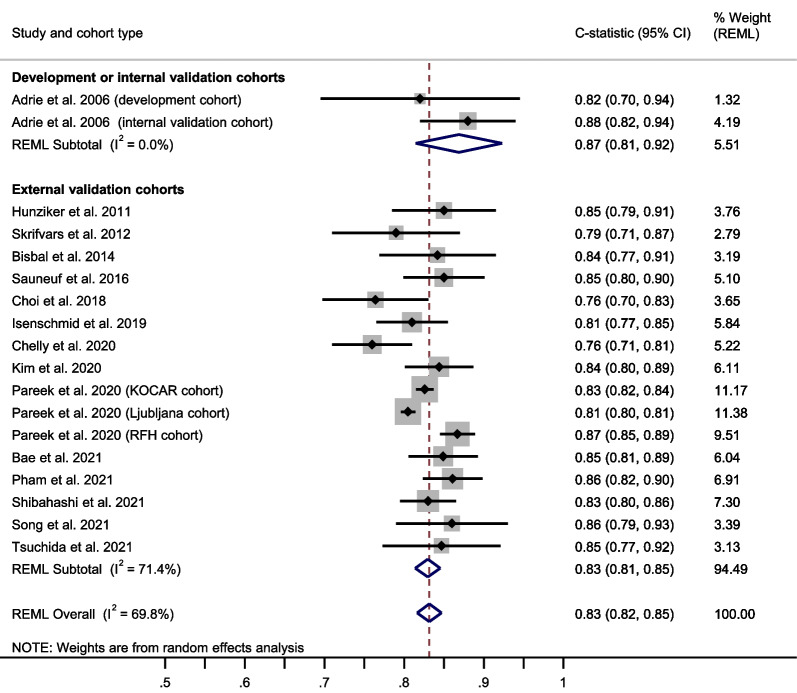
Meta-analysis of the C-statistic for the OHCA score. CI Confidence interval; KOCAR King’s Out-of-Hospital Cardiac Arrest Registry; OHCA Out-of-Hospital Cardiac Arrest; REML Restricted maximum likelihood; RFH Royal Free Hospital London

**Fig. 3:**
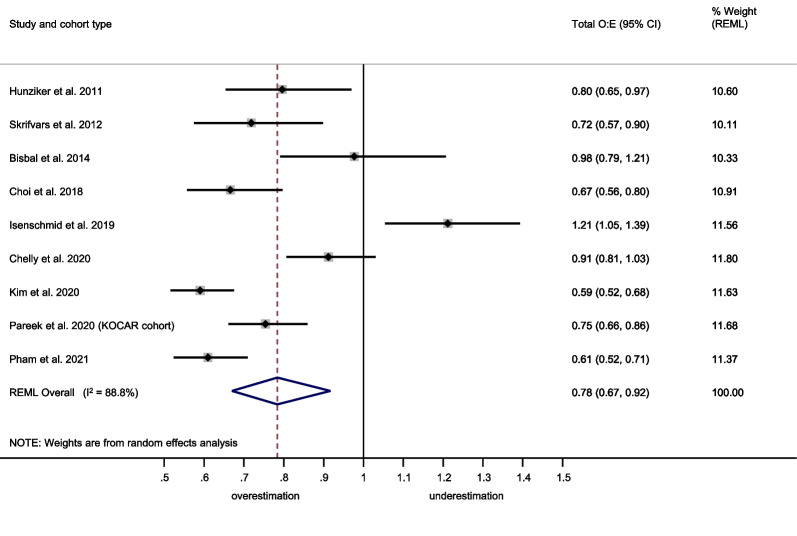
Meta-analysis of the total observed versus expected (O:E) ratio for the OHCA score. CI Confidence Interval; KOCAR King’s Out-of-Hospital Cardiac Arrest Registry; OHCA Out-of-Hospital Cardiac Arrest; REML Restricted maximum likelihood

The OHCA score showed a summary C-statistic of 0.83 (95% confidence interval [CI] 0.81–0.85) across 16 external validation cohorts[31, 32, 34, 35, 50–59] (**Fig. ****2**). For a meta-analysis of total O:E ratios, nine studies[31, 32, 34, 35, 50, 52–54, 56] provided sufficient data. The summary total O:E ratio was 0.78 (95% CI 0.67–0.92), showing an overestimation of poor outcome by the OHCA score (Fig. 3). For meta-analysis of the stratum-specific likelihood ratios, the number of studies reporting classification measures at the same score cut-offs as the original publication was too small.

### Prognostic performance of the CAHP score

**Fig. 4:**
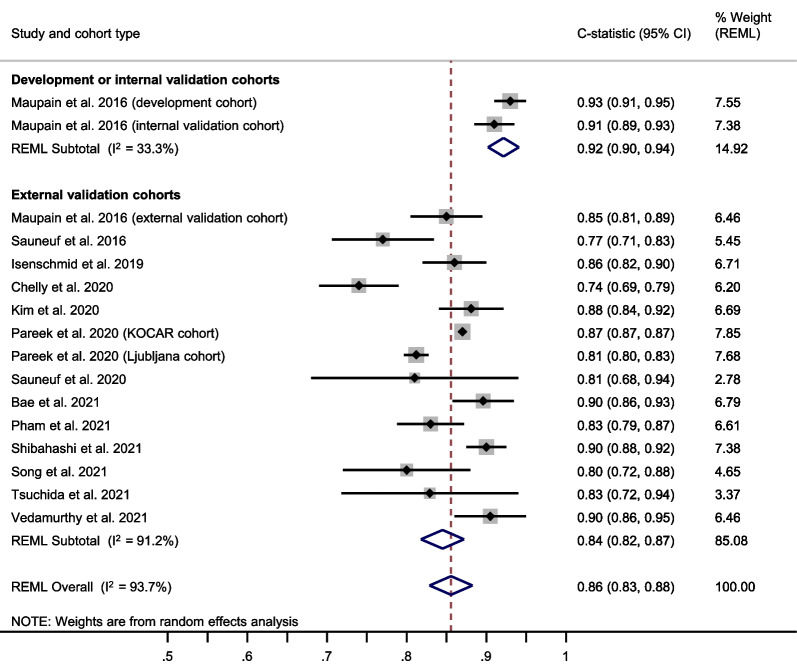
Meta-analysis of the C-statistic for the CAHP score. CAHP Cardiac Arrest Hospital Prognosis; CI Confidence Interval; KOCAR King’s Out-of-Hospital Cardiac Arrest Registry; REML Restricted maximum likelihood

**Fig. 5:**
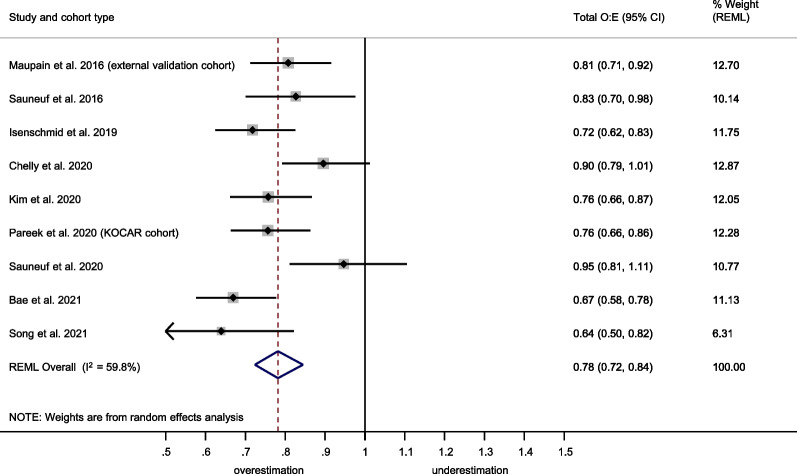
Meta-analysis of the total observed vs. expected (O:E) ratio for the CAHP score. CAHP Cardiac Arrest Hospital Prognosis; CI Confidence Interval; KOCAR King’s Out-of-Hospital Cardiac Arrest Registry; REML Restricted maximum likelihood

The CAHP score showed a summary C-statistic of 0.84 (95% CI 0.82–0.87) across 14 external validation cohorts[14, 35, 38, 51–59, 61] (Fig. 4). For a meta-analysis of total O:E ratios, nine studies[14, 35, 38, 51–55, 58] provided sufficient data. The summary total O:E ratio was 0.78 (95% CI 0.72–0.84), showing an overestimation of poor outcome by the CAHP score (Fig. 5). The stratum-specific likelihood ratios for poor neurological outcome in the low-risk, moderate-risk, and high-risk categories of the CAHP score were 0.21 (95% CI 0.18–0.26), 2.22 (95% CI 1.71–2.88) and 12.43 (95% CI 5.41–28.56) respectively (Additional file 1: Figure S1).

### Prognostic performance of the GO-FAR score

**Fig. 6:**
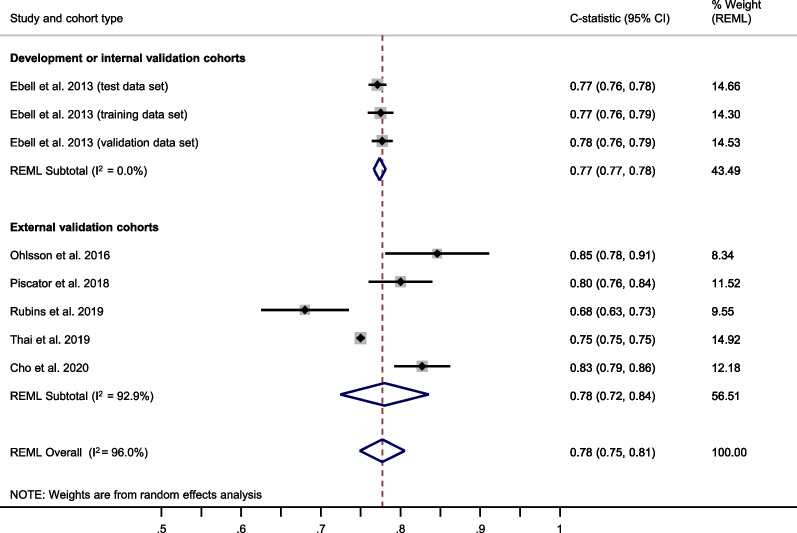
Meta-analysis of the C-statistic for the GO-FAR score. CI Confidence interval; GO-FAR Good Outcome Following Attempted Resuscitation; REML Restricted maximum likelihood

**Fig. 7:**
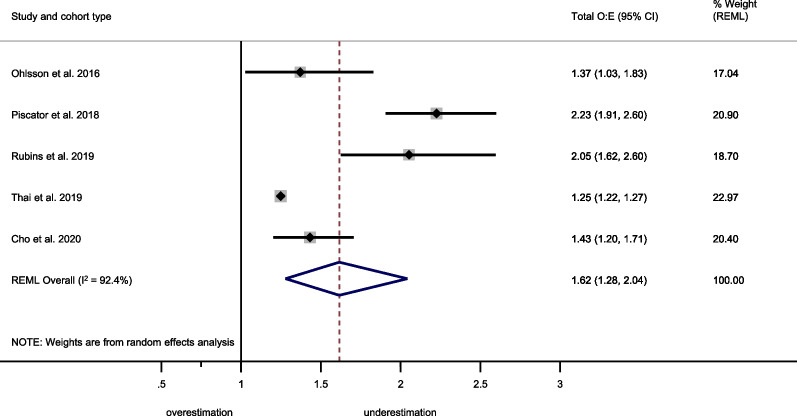
Meta-analysis of the total observed vs. expected (O:E) ratio for the GO-FAR score. CI Confidence interval; GO-FAR Good Outcome Following Attempted Resuscitation; REML Restricted maximum likelihood

The GO-FAR score showed a summary C-statistic of 0.78 (95% CI 0.72–0.84) across five external validation cohorts[40–42, 62, 63] (Fig. 6). Five studies[40–42, 62, 63] provided sufficient data for a meta-analysis of total O:E ratios. The summary total O:E ratio was 1.62 (95% CI 1.28–2.04), showing an underestimation of good outcome by the GO-FAR score (Fig. 7). For meta-analysis of the stratum-specific likelihood ratios, the number of studies reporting classification measures at the same score cut-offs as the original publication was too small.

### Subgroup analyses

Subgroup analyses were conducted to address heterogeneity in the inclusion criteria and in the outcome assessment across the validation studies of the OHCA and CAHP scores. Both scores showed good discriminatory performance across all subgroups with summary C-statistics being in the range of 0.80 to 0.85. The results are summarized in Additional file 1: Table S3 and shown in more detail in Additional file 1: Figures S2 to S6.

Sensitivity analyses with exclusion of studies with a high risk of bias were not conducted since only two studies were judged to have low risk of bias overall. Instead, a subgroup analysis was conducted, assessing the score performance in studies at high risk of bias due to the inclusion of an inappropriately small number of patients and in studies with an adequately large sample. The OHCA and CAHP scores performed similarly in both subgroups (Additional file 1: Table S3).

A random-effects meta-regression analysis showed no significant correlation between either mean/median patient age or percentage of a good outcome and C-statistic in the validation studies for the OHCA and CAHP score (Additional file 1: Figures S7 and S8). For the GO-FAR score, the number of studies was too small to perform subgroup and meta-regression analyses.

## Discussion

This systematic review and meta-analysis included results from 25 studies with a total of 124′168 patients to assess the prognostic performance of the three most thoroughly validated CPM for the prediction of mortality or poor neurological outcome after cardiac arrest. The analysis of the pooled data showed good discriminatory performance of the two post-arrest scores OHCA and CAHP with both scores performing similarly. An analysis of the overall calibration showed a slight overestimation of poor outcome for both scores.

The pre-arrest GO-FAR score showed acceptable discriminatory performance with the analysis of overall calibration showing substantial underestimation of good outcome.

Results were similar across all subgroup analyses, indicating that the results presented in this meta-analysis are robust and the OHCA and CAHP scores perform well in predicting mortality or neurological outcome as measured by CPC, with predictions being accurate for outcomes assessed at hospital discharge as well as up to 6 months after cardiac arrest. Meta-regressions of C-statistics for the OHCA and CAHP score showed that neither mean patient age nor percentage of observed good outcome correlates with the value of the C-statistic in the validation studies, indicating that these two scores perform well across different populations.

An important finding of this systematic review is the observed poor reporting across validation studies, a problem that has previously been highlighted [12, 67]. Especially calibration measures were found to be frequently missing, and if reported, the choice of reported measures was inconsistent.

There is an abundance of literature concerning CPM to predict outcomes in patients after cardiac arrest. A survey of available CPM identified 81 different prognostic models [12]. The authors found that novel CPM usually performed very good compared with established CPM used in other areas, indicating the potential of prognostic models in the prediction of outcomes after cardiac arrest [12]. However, only four of the 81 CPM have been validated more than twice resulting in the OHCA, CAHP, and GO-FAR score being the most thoroughly validated CPM [12]. However, the authors did not perform a formal meta-analysis of the CPM’s prognostic performance [12].

A recent systematic review by Gue et al.[68] aimed to summarise available clinical risk scores and their performance in a similar way, but only included scores with survival as the predicted outcome, as the authors deemed neurological outcome to be too ambiguous [68]. Eleven scoring systems predicting mortality after OHCA were identified and their development, calculation and performance summarised briefly. However, a meta-analysis of score performance measures was not conducted. The authors concluded that the scores with the most potential for clinical usefulness are the OHCA, revised post-cardiac arrest syndrome for therapeutic hypothermia (rCAST) and NULL-PLEASE scores [68]. The rCAST score was developed for use in patients treated with targeted temperature management only[69] and was therefore not included in our meta-analysis. The NULL-PLEASE score[70] is a CPM very similar to the OHCA and CAHP scores and has shown potential in some validation studies [56, 59, 71, 72].

A systematic review and meta-analysis assessed definitions of medical futility regarding CPR and the predictive value of pre-arrest risk scores including the GO-FAR score [17]. In a meta-analysis, a GO-FAR score of 14 points or higher predicted poor neurological outcome including death defined as a CPC score of ≥ 2 with a pooled specificity of 95%. However, a meta-analysis of C-statistics and calibration was not conducted.

The OHCA and CAHP scores have been criticised as being too difficult to calculate and therefore impractical to use in daily clinical practice [55]. However, there are now online calculators available, which render the calculation of these scores straightforward and easy [73–75]. Nevertheless, the issue of frequently missing or inadequately reported no-flow intervals remains. Especially in cases of unwitnessed cardiac arrest, no-flow times frequently cannot be reconstructed. In reaction to this criticism, an interesting novel CPM has been developed recently: The PROLOGUE score [55]. This CPM does not use the no-flow interval but instead includes two clinical neurological variables, namely the presence or absence of the pupillary light reflex and the GCS motor score, both evaluated at hospital admission. The other variables are similar to the OHCA and CAHP score’s with some different laboratory parameters included. In the internal validation data set, it showed an excellent C-statistic of 0.94 [55], but so far no external validations have been conducted.

A promising alternative to the development of ever more novel CPM is to improve established scoring systems by modifying them. Some studies tried to simplify them by omitting difficult to obtain parameters (e.g., no-flow interval) from the calculation [57], others added variables such as laboratory parameters (e.g., neuron-specific enolase), electroencephalography or imaging findings, or clinical parameters (e.g., GCS motor score) with promising results [37, 53, 58]. By modifying established scores, such as the OHCA, CAHP and GO-FAR scores according to recent scientific evidence and subsequently validating them, their predictive value and usability could be further enhanced and updated.

This review has limitations. First, as calibration measures were frequently missing, corresponding calibration measures had to be approximated. Although evidence-based approaches were used [45, 46], the results have to be interpreted cautiously. Furthermore, it is essential to note that the total O:E ratio only gives a rough overall estimate of calibration. Substantial miscalibration in specific risk strata might remain undetected [76, 77]. For example, validation studies presenting calibration plots usually found underestimation of poor outcome in low-risk categories, but good calibration in the high-risk categories for the OHCA score[31, 57]. Before use within a particular population, a validation study and, in case of miscalibration, model updating with re-calibration is recommended [26, 77]. Second, a majority of the included studies were rated as being at a high risk of bias, limiting our results' external validity and generalisability. Third, the present study focused on the OHCA, CAHP, and GO-FAR scores as it was not the aim to provide a systematic review of all available CPM in patients with cardiac arrest, but to assess the prognostic performance of the most thoroughly validated models as compiled by a previous systematic review [12]. Still, this might have resulted in selection bias.

A general limitation of prognostic factor research is the effect of self-fulfilling prophecy [78–80]. The concern is, that the sheer documentation of poor prognosis as such leads to a higher probability of poor outcome for the respective patient group as it might influence the treating physicians in their decision-making and may thus lead to a premature withdrawal of life-sustaining therapies [78–80]. However, to overcome this problem, treating physicians would have to be blinded with regard to all predictive factors necessary to calculate the CPM assessed in the respective study. These factors usually include clinical and laboratory parameters essential for clinical decision-making and thus cannot be withheld. Therefore, a certain risk of self-fulfilling prophecy is imminent to prognostic factor and prediction model studies.

On the other hand, this systematic review and meta-analysis has several strengths. First, it followed a strict methodology explicitly developed for prognostic model research [26, 28, 45]. Second, the robustness of our data was proven by various subgroup analyses, which means that it provides reliable evidence about the performance of three important cardiac arrest scores. Third, the present work compiles evidence from 25 observational studies with a total of 124′168 cardiac arrest patients underlining its statistical power and external validity.

## Conclusion

The OHCA and CAHP scores show good prognostic accuracy in predicting poor neurological outcome or mortality in patients after cardiac arrest and may help to support early discussions concerning goals of care and the extent of therapeutic effort. The GO-FAR score shows acceptable performance in predicting the chances of survival with good neurological outcome in case of an in-hospital cardiac arrest in a pre-arrest setting and could be a useful tool in code status discussions. Future predictive research studies should follow current methodological and reporting guidelines to ensure the validity and usability of their results [26, 28, 81, 82].

## Supplementary Information

**Additional file 1:** Full search strategy, risk of bias assessment and results of subgroup analyses.

## Data Availability

The datasets used and/or analysed during the current study are available from the corresponding author on reasonable request.

## Abbreviations

ICU: Intensive care unit
WLST: Withdrawal of life-sustaining therapy
ROSC: Return of spontaneous circulation
CPM: Clinical prediction model
CAHP: Cardiac arrest hospital prognosis
OHCA: Out-of-hospital cardiac arrest
CPC: Cerebral performance category scale
IHCA: In-hospital cardiac arrest
GO-FAR: Good outcome following attempted resuscitation
CPR: Cardiopulmonary resuscitation
CHARMS: Critical appraisal and data extraction for systematic reviews of prediction modelling studies
PRISMA: Preferred reporting items for systematic reviews and meta-analyses
MOOSE: Meta-analysis of observational studies in epidemiology
PROSPERO: International prospective register of systematic reviews
AUROC: Area under the receiver operating characteristic curve
O:E: Observed versus expected ratio
ROB: Risk of bias
PROBAST: Prediction model risk of bias assessment tool
REML: Restricted maximum likelihood
rCAST: Revised post-cardiac arrest syndrome for therapeutic hypothermia score
PROLOGUE: Prognostication using logistic regression model for unselected adult cardiac arrest patients in the early stages
