# Supplementary material for: Predicting neurological outcome in adult patients with cardiac arrest: systematic review and meta-analysis of prediction model performance
Source: Crit Care. 2022 Dec 11;26:382. doi: 10.1186/s13054-022-04263-y (PMC9741710; doi:10.1186/s13054-022-04263-y)
Supplement: Supplementary file 1 — Additional file 1: Full search strategy, risk of bias assessment and results of subgroup analyses. [file 13054_2022_4263_MOESM1_ESM.pdf]

SUPPLEMENTARY FILE

Full Search Strategy

Embase.com

(20211207; 366 hits)

('good outcome following attempted resuscitation score'/exp OR 'go far score'/exp OR 'cardiac arrest hospital prognosis score'/exp OR 'out of hospital cardiac arrest score'/exp OR ('Good Outcome Following Attempted Resuscitation' OR GO-FAR OR 'Cardiac Arrest Hospital Prognosis' OR CAHP OR (('out of hospital cardiac arrest' OR OHCA OR sOHCA) NEAR/3 scor*)):ab,ti,kw)

AND

[1-11-2006]/sd

NOT (juvenile/exp NOT adult/exp) NOT (('animal'/de OR 'animal experiment'/exp OR 'nonhuman'/de) NOT ('human'/exp OR 'human experiment'/de)) NOT [conference abstract]/lim

Ovid Medline

(20211207; Ovid MEDLINE(R) ALL 1946 to December 06, 2021; 370 hits)

(Good Outcome Following Attempted Resuscitation OR GO-FAR OR Cardiac Arrest Hospital Prognosis OR CAHP OR ((out of hospital cardiac arrest OR OHCA OR sOHCA) ADJ3 scor*)).ab,ti,kf.

NOT ((exp adolescent/ OR exp child/ OR exp infant/) NOT exp adult/) NOT (exp animals/ NOT humans/)

limit 1 to dt=20061101-20211231

Web of Science Core Collection

(20211207; Editions = A&HCI, BKCI-SSH, BKCI-S, CCR-EXPANDED, ESCI, IC, CPCI-SSH, CPCI-S, SCI-EXPANDED, SSCI; 122 hits)

TS=(("Good Outcome Following Attempted Resuscitation" OR "Cardiac Arrest Hospital Prognosis" OR CAHP OR (("out of hospital cardiac arrest" OR OHCA OR sOHCA) NEAR/2 scor*)))

AND

DOP=2006-11-01/2021-12-31

Search narrative

- OHCA as a score name alone could not be searched because of the many papers referring to OHCA as an indication. For this reason, OHCA was combined with a proximity operator to the word scor*.
- GO-FAR searched as a text word gives a considerable background noise. We have decided to keep the acronym GO-FAR for the searches in biomedical databases (Embase and Medline), but to remove it from the Web of Science search. There, it produced a background noise of several thousand hits.

Supplementary Tables and Figures

Supplementary Table 1 – Risk of bias and applicability of eligible studies

		Risk of bias				Applicability			Overall	
Author	Year	Participants	Predictors	Outcome	Analysis	Participants	Predictors	Outcome	ROB	Applicability
**OHCA score**										
Adrie et al. (development)	2006	+	+	+	-	+	+	+	-	+
Adrie et al. (validation)	2006	+	+	+	-	+	+	+	-	+
Hunziker et al.	2011	+	+	+	-	+	+	+	-	+
Skrifvars et al.	2012	+	+	+	-	+	+	+	-	+
Bisbal et al.	2014	+	+	+	-	+	+	+	-	+
Sauneuf et al.	2016	+	+	+	-	+	+	+	-	+
Choi et al.	2018	+	+	+	-	+	+	+	-	+
Isenschmid et al.	2018	+	+	+	-	+	+	+	-	+
Chelly et al.	2020	+	+	+	-	+	+	+	-	+
Kim et al.	2020	+	+	+	-	+	+	+	-	+
Pareek et al. (KOCAR cohort)	2020	+	+	+	-	+	+	+	-	+
Pareek et al. (Ljubljana cohort)	2020	+	+	+	-	+	+	+	-	+
Pareek et al. (RFH cohort)	2020	+	+	+	-	+	+	+	-	+
Bae et al.	2021	+	+	+	-	+	+	+	-	+
Pham et al.	2021	+	+	+	-	+	+	+	-	+
Shibahashi et al.	2021	+	+	+	+	+	+	+	+	+
Song et al.	2021	+	+	+	-	+	+	+	-	+
Tsuchida et al.	2021	+	+	+	-	+	+	+	-	+
**CAHP score**										
Maupain et al. (development)	2016	+	+	+	-	+	+	+	-	+
Maupain et al. (validation)	2016	+	+	+	-	+	+	+	-	+

Sauneuf et al.	2016	+	+	+	-	+	+	+	-	+
Isenschmid et al.	2018	+	+	+	-	+	+	+	-	+
Chelly et al.	2020	+	+	+	-	+	+	+	-	+
Kim et al.	2020	+	+	+	-	+	+	+	-	+
Pareek et al. (KOCAR cohort)	2020	+	+	+	-	+	+	+	-	+
Pareek et al. (Ljubljana cohort)	2020	+	+	+	-	+	+	+	-	+
Pareek et al. (RFH cohort)	2020	+	+	+	-	+	+	+	-	+
Sauneuf et al.	2020	+	+	+	-	+	+	+	-	+
Bae et al.	2021	+	+	+	-	+	+	+	-	+
Pham et al.	2021	+	+	+	-	+	+	+	-	+
Shibahashi et al.	2021	+	+	+	+	+	+	+	+	+
Song et al.	2021	+	+	+	-	+	+	+	-	+
Tsuchida et al.	2021	+	+	+	-	+	+	+	-	+
Vedamurthy et al.	2021	+	+	+	-	+	+	+	-	+
**GO-FAR score**										
Ebell et al. (training data set)	2013	+	+	+	-	+	+	+	-	+
Ebell et al. (test data set)	2013	+	+	+	-	+	+	+	-	+
Ebell et al. (validation data set)	2013	+	+	+	-	+	+	+	-	+
Ohlsson et al.	2016	+	+	+	-	+	+	+	-	+
Piscator et al.	2018	+	+	+	+	+	+	+	+	+
Rubins et al.	2019	+	+	+	-	+	+	+	-	+
Thai et al.	2019	+	+	+	-	+	+	+	-	+
Cho et al.	2020	+	+	+	-	+	+	+	-	+
Aldabagh et al.	2021	+	+	+	-	+	+	+	-	+

+ indicates low risk of bias/high applicability; - indicates high risk of bias/low applicability. **GO-FAR** Good Outcome Following Attempted Resuscitation; **CAHP** Cardiac Arrest Hospital Prognosis; **KOCAR** King's Out of Hospital Cardiac Arrest Registry; **OHCA** Out-of-Hospital Cardiac Arrest; **RFH** Royal Free Hospital London; **ROB** Risk of bias.

Supplementary Table 2 – Detailed risk of bias rating for the domain «analysis»										
**Signalling question**		4.1 Reasonable number of participants with the outcome	4.2 Appropriate handling of continuous and categorical predictors	4.3 Inclusion of all enrolled participants into the analysis	4.4 Missing data handled by multiple imputation	4.5 Avoidance of predictor selection based on univariable analysis	4.6 Appropriate accountance of complexities in data	4.7 Appropriate evaluation of calibration and discrimination	4.8 Accountance for overfitting and/or optimism	4.9 Correspondence of assigned weight of predictors with results from the multivariable analysis
**OHCA score**										
Adrie et al. (development cohort)	2006	No	Yes	Yes	NI	No	Yes	No	No	Yes
Adrie et al. (validation cohort)	2006	No	Yes	Yes	NI	NA	Yes	Yes	NA	NA
Hunziker et al.	2011	No	Yes	No	No	NA	Yes	Yes	NA	NA
Skrifvars et al.	2012	No	Yes	Yes	No	NA	Yes	No	NA	NA
Bisbal et al.	2014	No	Yes	Yes	No	NA	Yes	No	NA	NA
Sauneuf et al.	2016	No	Yes	Yes	NI	NA	Yes	No	NA	NA
Choi et al.	2018	No	Yes	Yes	No	NA	Yes	No	NA	NA
Isenschmid et al.	2018	Yes	Yes	Yes	Yes	NA	Yes	No	NA	NA
Chelly et al.	2020	Yes	Yes	No	No	NA	Yes	No	NA	NA
Kim et al.	2020	Yes	Yes	NI	NI	NA	Yes	No	NA	NA
Pareek et al. (KOCAR cohort)	2020	Yes	Yes	No	Yes	NA	Yes	Yes	NA	NA

Pareek et al. (Ljubljana cohort)	2020	Yes	Yes	No	Yes	NA	Yes	Yes	NA	NA
Pareek et al. (RFH cohort)	2020	No	Yes	No	Yes	NA	Yes	Yes	NA	NA
Bae et al.	2021	NI	Yes	No	No	NA	Yes	No	NA	NA
Pham et al.	2021	Yes	Yes	Yes	No	NA	Yes	No	NA	NA
Shibahashi et al.	2021	Yes	Yes	Yes	Yes	NA	Yes	Yes	NA	NA
Song et al.	2021	No	Yes	Yes	No	NA	Yes	No	NA	NA
Tsuchida et al.	2021	No	Yes	No	No	NA	Yes	No	NA	NA
**CAHP score**										
Maupain et al. (development cohort)	2016	Yes	Yes	No	No	No	Yes	No	No	Yes
Maupain et al. (internal and external validation)	2016	Yes	Yes	No	No	NA	Yes	No	NA	NA
Sauneuf et al.	2016	No	Yes	No	Yes	NA	Yes	No	NA	NA
Isenschmid et al.	2018	Yes	Yes	Yes	Yes	NA	Yes	No	NA	NA
Chelly et al.	2020	Yes	Yes	No	No	NA	Yes	No	NA	NA
Kim et al.	2020	Yes	Yes	NI	NI	NA	Yes	No	NA	NA
Pareek et al. (KOCAR cohort)	2020	Yes	Yes	No	Yes	NA	Yes	Yes	NA	NA
Pareek et al. (Ljubljana cohort)	2020	Yes	Yes	No	Yes	NA	Yes	Yes	NA	NA
Pareek et al. (RFH cohort)	2020	No	Yes	No	Yes	NA	Yes	Yes	NA	NA
Sauneuf et al.	2020	Yes	Yes	No	No	NA	Yes	No	NA	NA
Bae et al.	2021	NI	Yes	No	No	NA	Yes	No	NA	NA
Pham et al.	2021	Yes	Yes	Yes	No	NA	Yes	No	NA	NA
Shibahashi et al.	2021	Yes	Yes	Yes	Yes	NA	Yes	Yes	NA	NA
Song et al.	2021	No	Yes	Yes	No	NA	Yes	No	NA	NA

Tsuchida et al.	2021	No	Yes	No	No	NA	Yes	No	NA	NA
Vedamurthy et al.	2021	No	Yes	No	No	NA	Yes	No	NA	NA
**GO-FAR score**										
Ebell et al. 2013 (training data set)	2013	Yes	Yes	No	No	Yes	Yes	No	No	Yes
Ebell et al. 2013 (test data set)	2013	Yes	Yes	No	No	NA	Yes	Yes	NA	NA
Ebell et al. 2013 (validation data set)	2013	Yes	Yes	No	No	NA	Yes	No	NA	NA
Ohlsson et al. 2016	2016	No	Yes	Yes	Yes	NA	Yes	No	NA	NA
Piscator et al. 2018	2018	Yes	Yes	Yes	Yes	NA	Yes	Yes	NA	NA
Rubins et al. 2019	2019	No	Yes	Yes	Yes	NA	Yes	No	NA	NA
Thai et al. 2019	2019	Yes	Yes	No	No	NA	Yes	Yes	NA	NA
Cho et al. 2020	2020	Yes	Yes	Yes	NI	NA	Yes	No	NA	NA
Aldabagh et al. 2021	2021	No	Yes	Yes	NI	NA	Yes	No	NA	NA
**GO-FAR** Good Outcome Following Attempted Resuscitation; **CAHP** Cardiac Arrest Hospital Prognosis; **KOCAR** King's Out of Hospital Cardiac Arrest Registry; **NA** Not applicable; **NI** No information; **OHCA** Out-of-Hospital Cardiac Arrest; **RFH** Royal Free Hospital London.										

Supplementary Table 3 – Results of Subgroup Analysis

Analysis	Subgroup	n	C-statistic	Forest plot
**OHCA score**				
**Analysis**	**Subgroup**	**n**	**C-statistic**	**Forest plot**
Type of cardiac arrest	OHCA only	12	0.84 (0.82-0.85)	Suppl. Figure 2
	IHCA or mixed	4	0.80 (0.76-0.83)	
Assessed outcome	Neurological outcome	13	0.83 (0.81-0.85)	Suppl. Figure 3
	Mortality	3	0.84 (0.81-0.88)	
Time of outcome assessment	Hospital discharge to 1 month	13	0.82 (0.81-0.84)	Suppl. Figure 4
	> 1 to 6 months	3	0.85 (0.82-0.88)	
Sample size	Appropriate	7	0.82 (0.80-0.84)	Not shown
	Inappropriately small	9	0.84 (0.82-0.87)	
**CAHP score**				
**Analysis**	**Subgroup**	**n**	**C-statistic**	**Forest plot**
Type of cardiac arrest	OHCA only	12	0.85 (0.83-0.88)	Suppl. Figure 5
	IHCA or mixed	2	0.80 (0.68-0.92)	
Assessed outcome	Neurological outcome	13	0.84 (0.82-0.87)	Not shown
	Mortality	1	0.83 (0.79-0.87)	
Time of outcome assessment	Hospital discharge to 1 month	12	0.84 (0.81-0.88)	Suppl. Figure 6
	> 1 to 6 months	2	0.85 (0.78-0.91)	
Sample size	Appropriate	9	0.84 (0.81-0.87)	Not shown
	Inappropriately small	5	0.85 (0.79-0.90)	

Summary of pooled C-statistic of the separate subgroups. For more details see the forest plots below. **IHCA** In-Hospital Cardiac Arrest; **OHCA** Out-of-Hospital Cardiac Arrest.

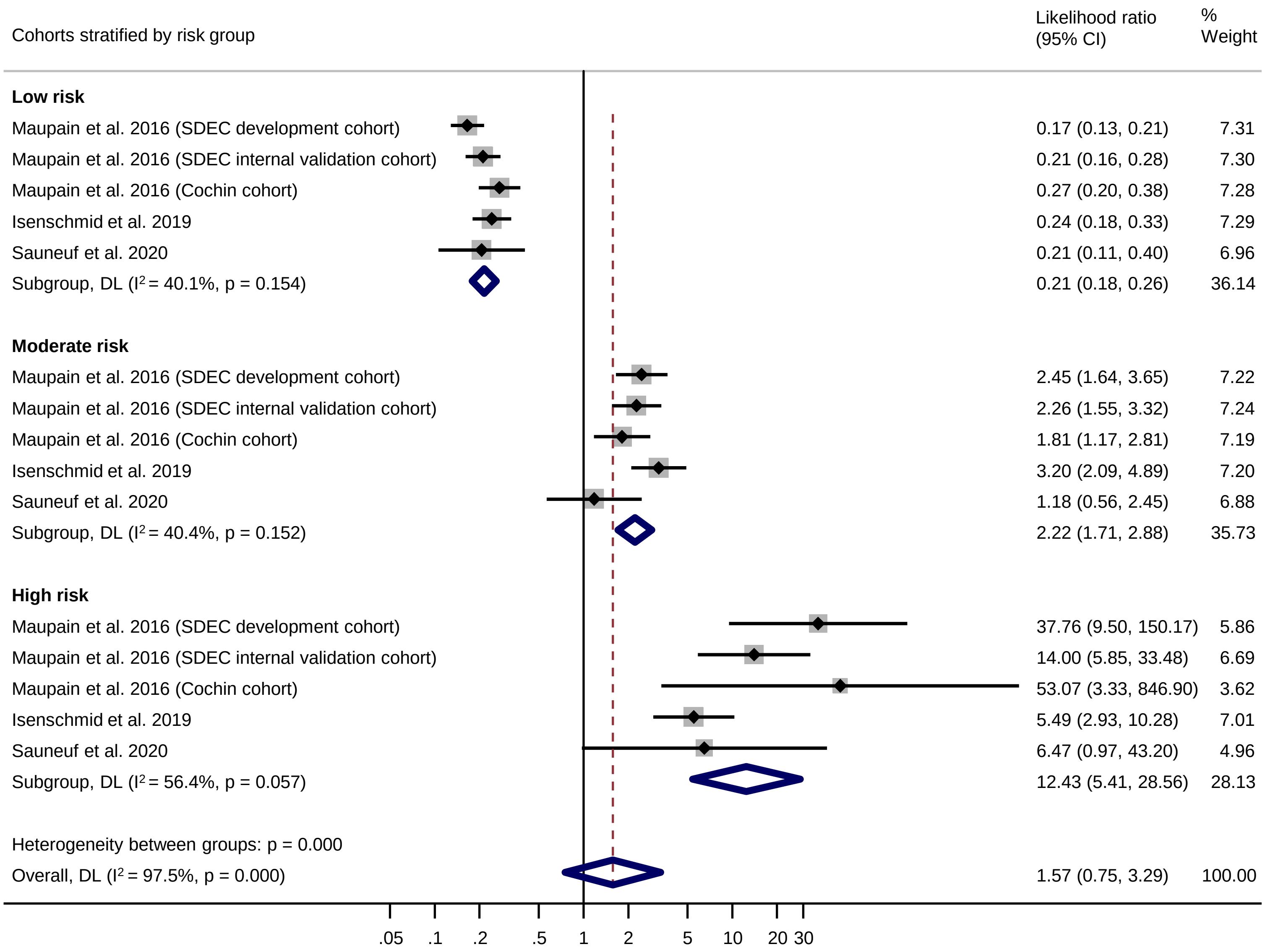

NOTE: Weights and between-subgroup heterogeneity test are from random-effects model; continuity correction applied to studies with zero cells

Supplementary Figure 1: Forest plot depicting pooled estimates of stratum-specific likelihood ratios for the three risk groups of the CAHP score. **CAHP** Cardiac Arrest Hospital Prognosis; **CI** Confidence interval; **DL** DerSimonian-Laird; **SDEC** Sudden Death Expert Registry.

Cohorts stratified by cardiac arrest setting	C-statistic (95% CI)	% Weight (REML)
**OHCA**		
Hunziker et al. 2011	0.85 (0.79, 0.91)	3.95
Sauneuf et al. 2016	0.85 (0.80, 0.90)	5.37
Choi et al. 2018	0.76 (0.70, 0.83)	3.83
Kim et al. 2020	0.84 (0.80, 0.89)	6.45
Pareek et al. 2020 (KOCAR cohort)	0.83 (0.82, 0.84)	11.92
Pareek et al. 2020 (Ljubljana cohort)	0.81 (0.80, 0.81)	12.16
Pareek et al. 2020 (RFH cohort)	0.87 (0.85, 0.89)	10.12
Bae et al. 2021	0.85 (0.81, 0.89)	6.38
Pham et al. 2021	0.86 (0.82, 0.90)	7.31
Shibahashi et al. 2021	0.83 (0.80, 0.86)	7.73
Song et al. 2021	0.86 (0.79, 0.93)	3.56
Tsuchida et al. 2021	0.85 (0.77, 0.92)	3.28
REML Subtotal (I^2 = 71.8%)	0.84 (0.82, 0.85)	82.07
**IHCA or mixed**		
Skrifvars et al. 2012	0.79 (0.71, 0.87)	2.92
Bisbal et al. 2014	0.84 (0.77, 0.91)	3.35
Isenschmid et al. 2019	0.81 (0.77, 0.85)	6.16
Chelly et al. 2020	0.76 (0.71, 0.81)	5.50
REML Subtotal (I^2 = 26.7%)	0.80 (0.76, 0.83)	17.93
REML Overall (I^2 = 71.4%)	0.83 (0.81, 0.85)	100.00

NOTE: Weights are from random effects analysis

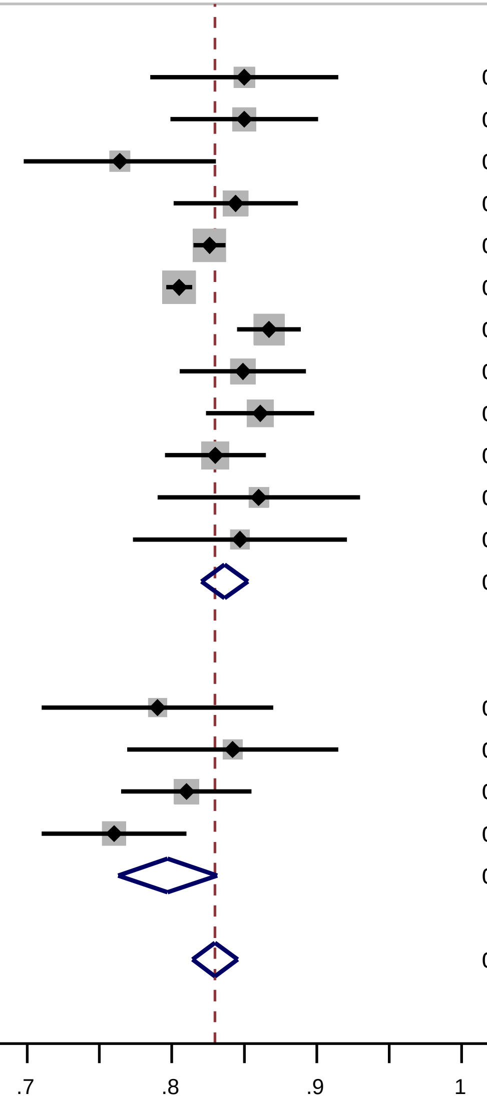

Supplementary Figure 2: Subgroup analysis comparing the performance of the OHCA score in cohorts with OHCA patients only vs. cohorts with IHCA patients only or mixed populations. **CI** Confidence interval; **IHCA** In-Hospital Cardiac Arrest; **KOCAR** King's Out-of-Hospital Cardiac Arrest Registry; **OHCA** Out-of-Hospital Cardiac Arrest; **REML** Restricted maximum likelihood; **RFH** Royal Free Hospital London.

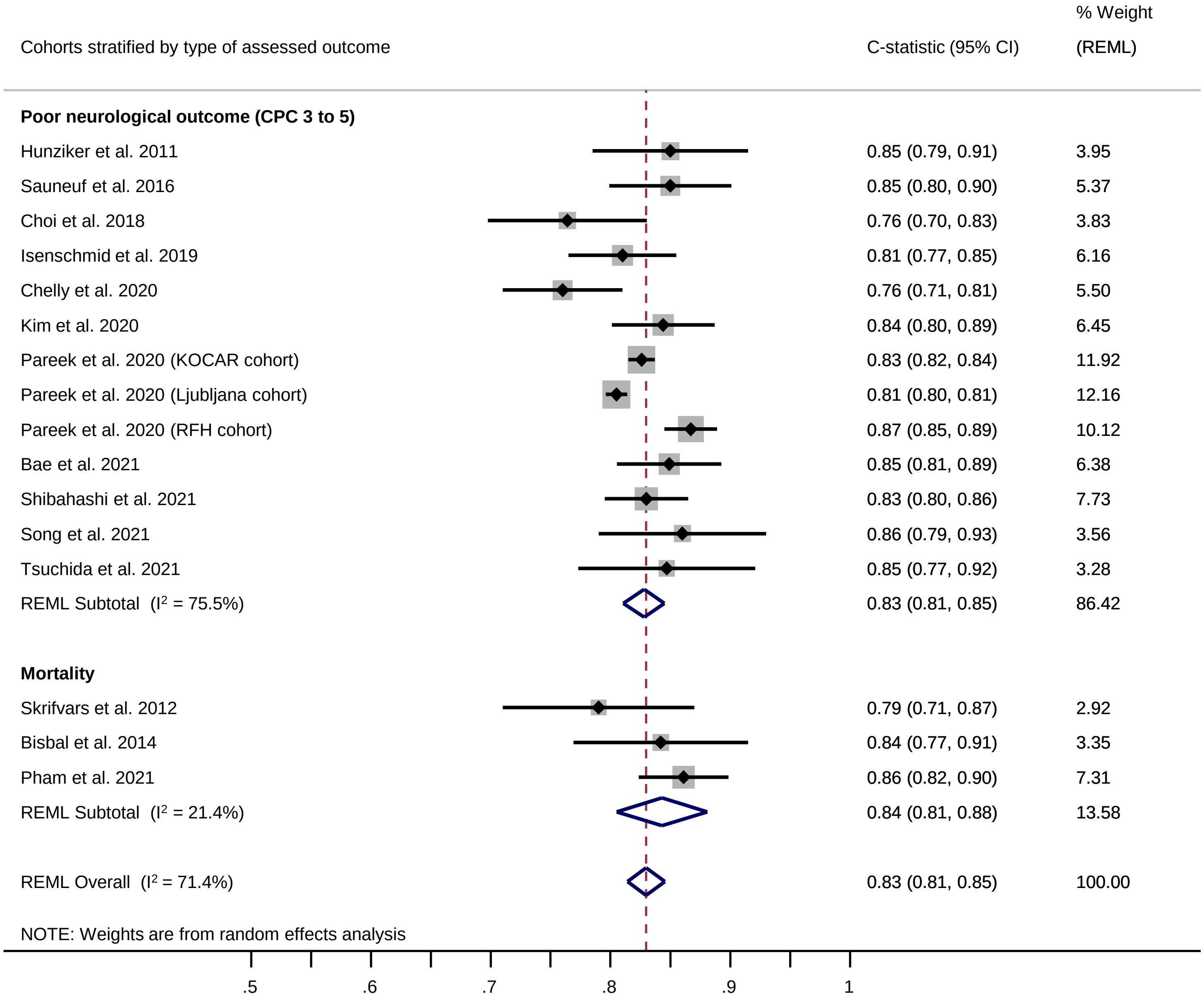

Supplementary Figure 3: Subgroup analysis comparing the performance of the OHCA score in cohorts with assessment of poor neurological outcome vs. cohorts with assessment of mortality only. **CI** Confidence interval; **CPC** Cerebral Performance Category scale; **KOCAR** King's Out-of-Hospital Cardiac Arrest Registry; **OHCA** Out-of-Hospital Cardiac Arrest; **REML** Restricted maximum likelihood; **RFH** Royal Free Hospital London.

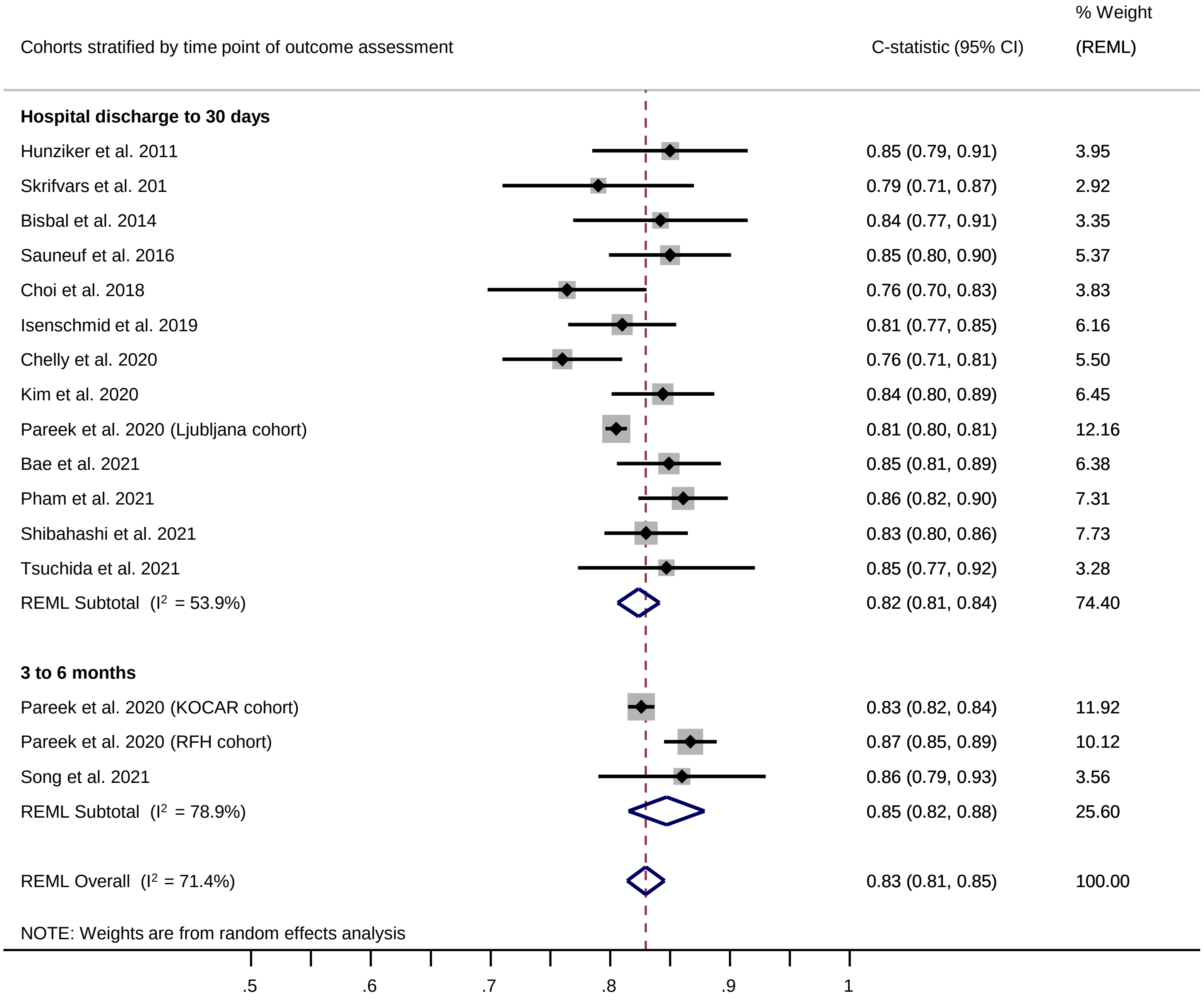

Supplementary Figure 4: Subgroup analysis comparing the performance of the OHCA score in cohorts with outcome assessment at hospital discharge to 30 days vs. cohorts with assessment of outcome at >1 month. **CI** Confidence interval; **KOCAR** King's Out-of-Hospital Cardiac Arrest Registry; **OHCA** Out-of-Hospital Cardiac Arrest; **REML** Restricted maximum likelihood; **RFH** Royal Free Hospital London.

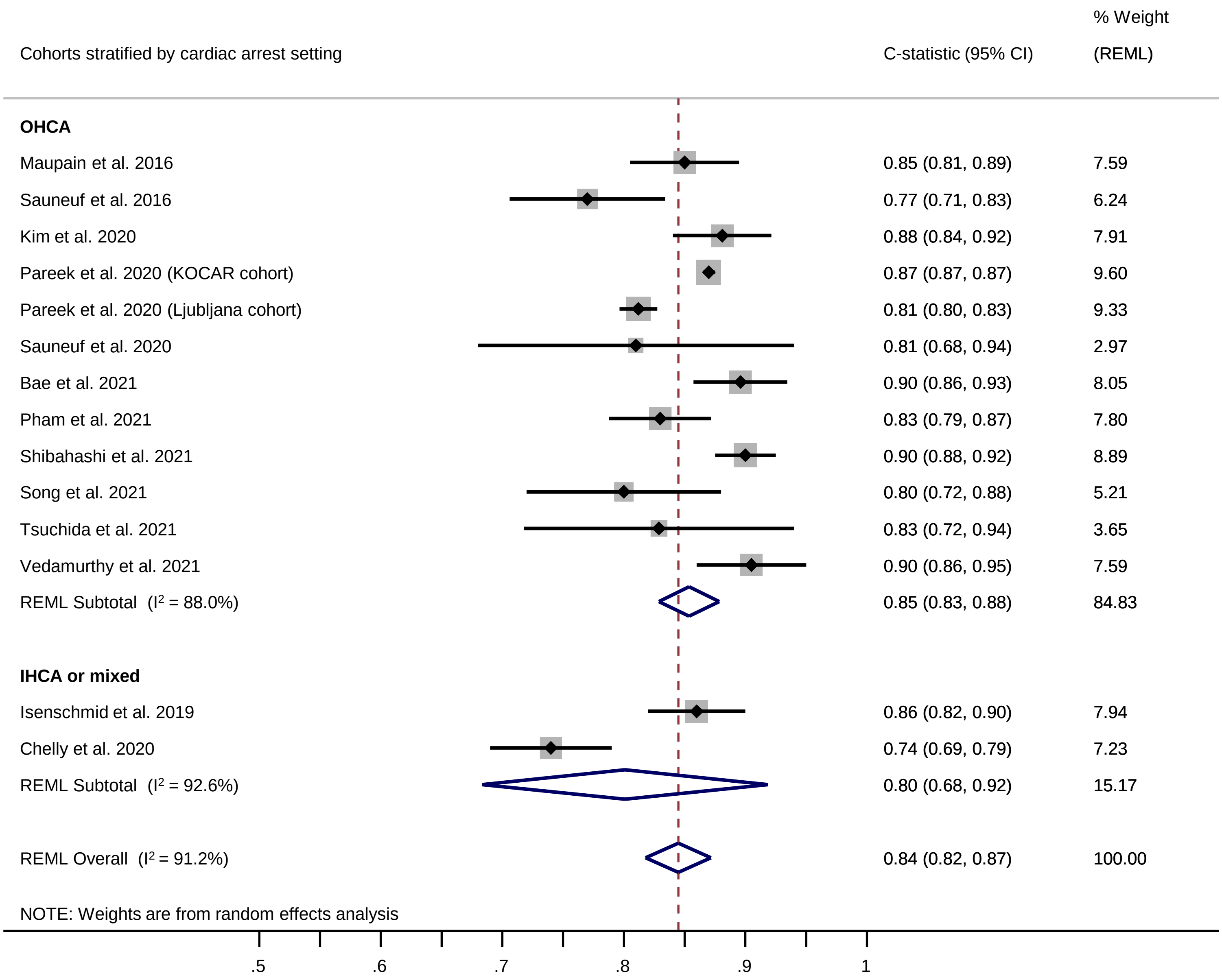

Supplementary Figure 5: Subgroup analysis comparing the performance of the CAHP score in cohorts with OHCA patients only vs. cohorts with IHCA patients only or mixed populations. **CAHP** Cardiac Arrest Hospital Prognosis; **CI** Confidence interval; **IHCA** In-Hospital Cardiac Arrest; **KOCAR** King's Out-of-Hospital Cardiac Arrest Registry; **OHCA** Out-of-Hospital Cardiac Arrest; **REML** Restricted maximum likelihood.

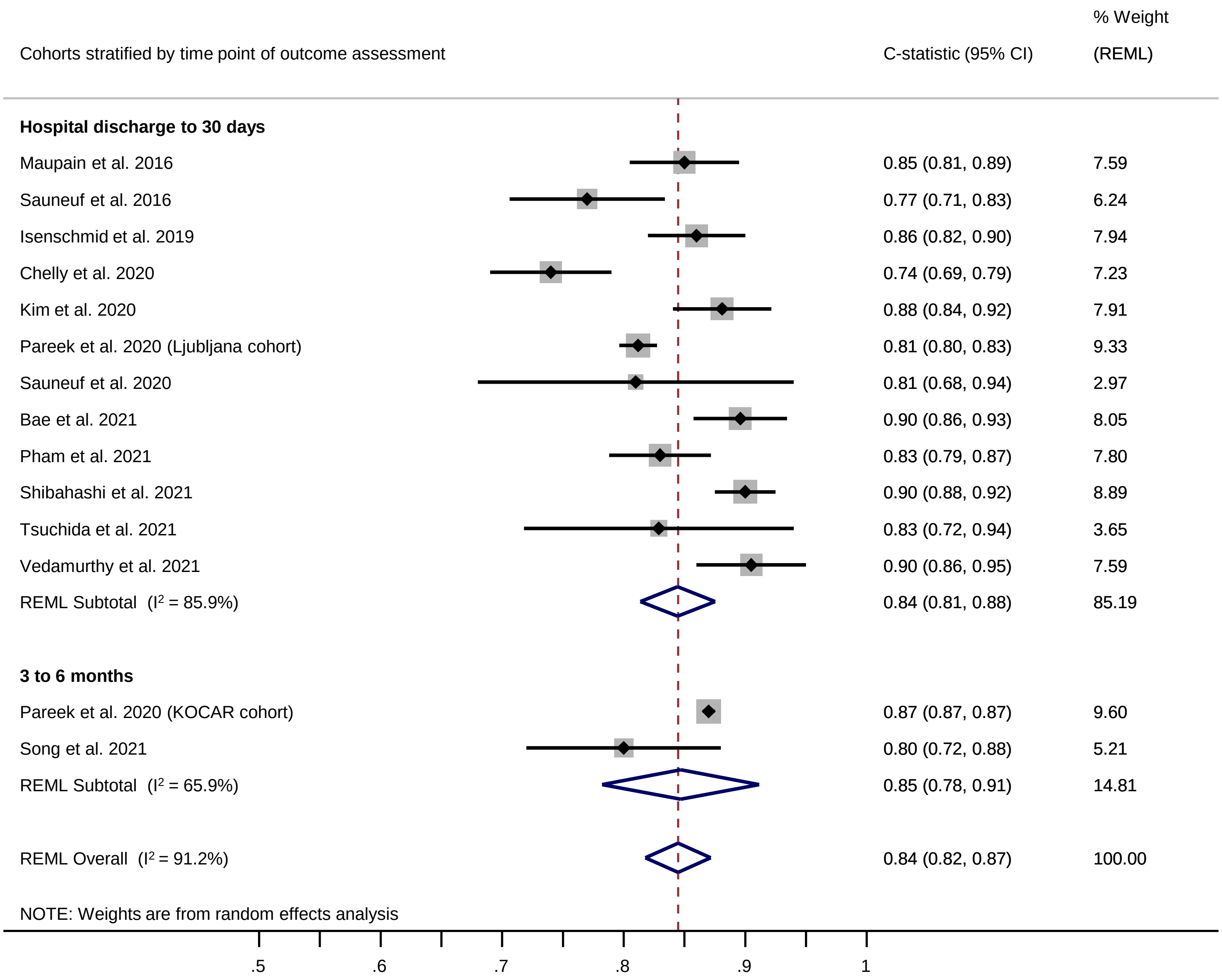

Supplementary Figure 6: Subgroup analysis comparing the performance of the CAHP score in cohorts with outcome assessment at hospital discharge to 30 days vs. cohorts with assessment of outcome at >1 month. **CAHP** Cardiac Arrest Hospital Prognosis; **CI** Confidence interval; **KOCAR** King's Out-of-Hospital Cardiac Arrest Registry; **REML** Restricted maximum likelihood.

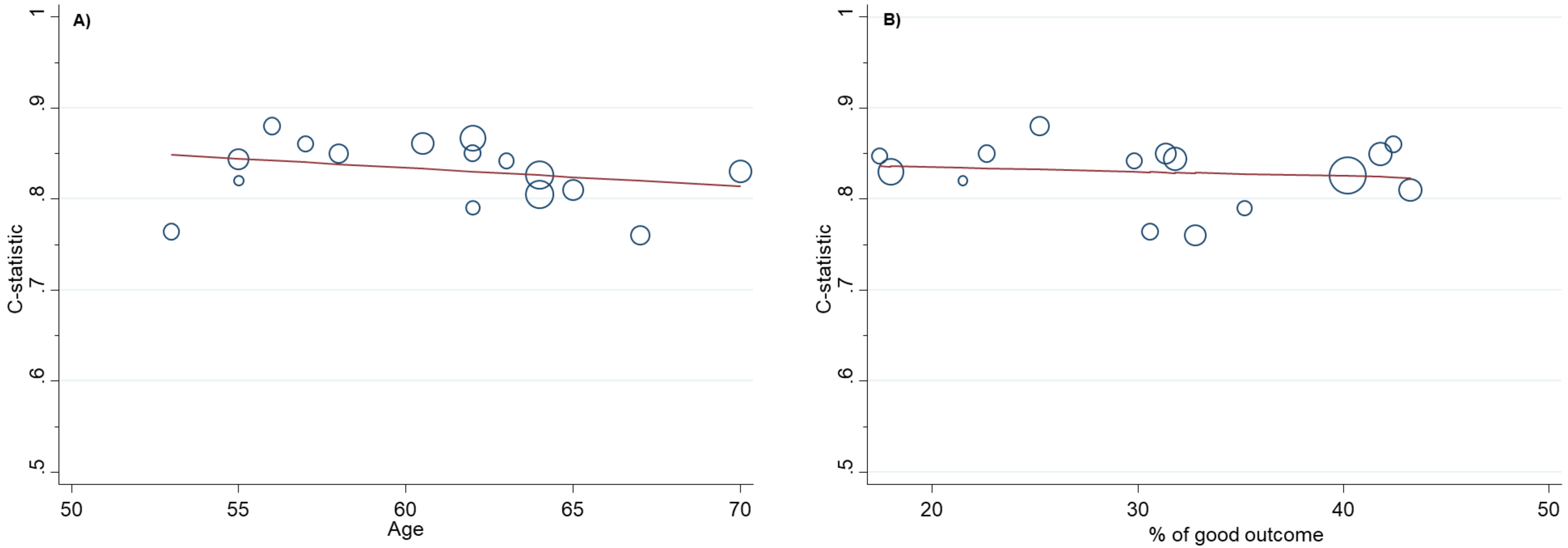

Supplementary Figure 7: Meta-regression analysis for the OHCA score. A) C-statistic vs. mean/median patient age. B) C-statistic vs. percentage of observed good outcome. **OHCA** Out-of-Hospital Cardiac Arrest.

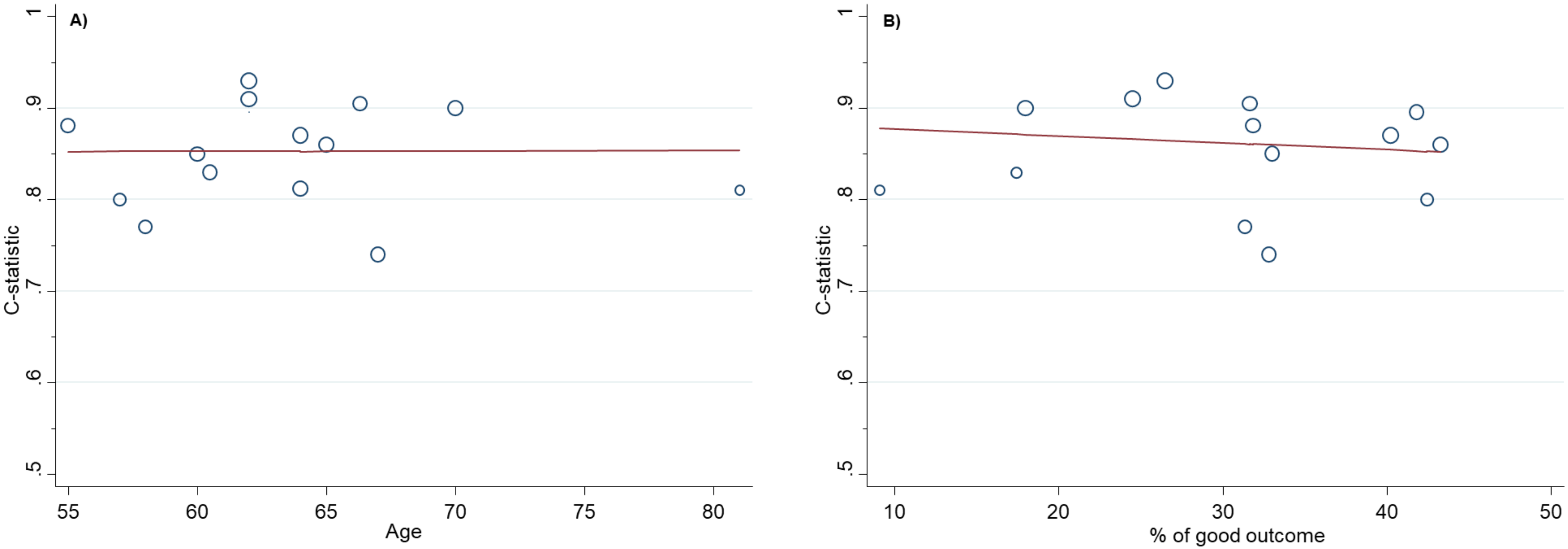

Supplementary Figure 8: Meta-regression analysis for the CAHP score. A) C-statistic vs. mean/median patient age. B) C-statistic vs. percentage of observed good outcome. **CAHP** Cardiac Arrest Hospital Prognosis.
